# The Relationship Between HIV Infection and Cardiovascular Disease

**DOI:** 10.2174/157340308785160589

**Published:** 2008-08

**Authors:** Birgitt Dau, Mark Holodniy

**Affiliations:** VA Palo Alto Health Care System and Division of Infectious Diseases and Geographic Medicine, Stanford University, Palo Alto, CA, USA

**Keywords:** HIV, cardiology, treatment, natural history, review.

## Abstract

Over 30 million people are currently living with human immunodeficiency virus (HIV) infection, and over 2 million new infections occur per year. HIV has been found to directly affect vascular biology resulting in an increased risk of cardiovascular disease compared to uninfected persons. Although HIV infection can now be treated effectively with combination antiretroviral medications, significant toxicities such as hyperlipidemia, diabetes, and excess cardiovascular co-morbidity; as well as the potential for significant drug-drug interactions between HIV and cardiovascular medications, present new challenges for the management of persons infected with HIV. We first review basic principles of HIV pathogenesis and treatment and then discuss relevant clinical management strategies that will be useful for cardiologists who might be involved in the care of HIV infected patients.

## INTRODUCTION TO HIV INFECTION

HIV-1 is an enveloped RNA retrovirus. HIV is transmitted by direct inoculation into the blood stream or after contact and attachment through mucosal surfaces. HIV preferentially infects CD4+ cells. HIV attaches to CD4 and other co-receptors such as CCR5 and CXCR4 on the surface of a cell in order to infect that cell. Within the cell, viral RNA is transcribed to DNA by the HIV reverse transcriptase. Viral DNA then migrates to the cell nucleus and integrates into the host cell genome utilizing the HIV integrase enzyme. The virus can remain latent, or using host factors, initiate viral transcription and translation. The HIV protease enzyme cleaves the viral polyprotein to form mature viral particles, which are then released from the cell to infect other cells. In the absence of HIV treatment, ongoing viral replication and infection of cells leads to CD4+ cell destruction and depletion resulting in immune system dysfunction. Once the CD4 cell count has decreased to < 200/mm^3^ or < 14%, HIV infection is known as the acquired immunodeficiency syndrome (AIDS). AIDS is manifested by development of opportunistic infections (OIs) and HIV-associated malignancies [1].

After infection the natural history of HIV disease can take several forms and is dependent on underlying host factors and virulence factors associated with different HIV strains. Most people usually have a non-specific viral illness after acute infection. For the majority of the HIV-infected population, a period of chronic (or clinically latent) infection can last years where the immune system is still able to function despite ongoing HIV replication and depletion of CD4 cells. Most HIV infected people require HIV treatment at some point during infection. A small percentage of those infected are able to control viral replication over many years in the absence of treatment and are referred to by various names (i.e., long term nonprogressors, elite controllers). Some develop AIDS relatively quickly and are referred to as rapid progressors. In the absence of HIV treatment, most HIV-infected persons with AIDS develop AIDS complications or death within 2-3 years.

UNAIDS and WHO estimate that approximately 33 million people are HIV infected world-wide and 2.5 million became infected and 2.1 million died of AIDS in 2007 [2]. The major risk factor worldwide for HIV infection is heterosexual contact. In the United States, approximately one million people are infected with HIV, and 25% don’t know they are HIV infected. It is also estimated that about 40,000 new HIV infections occur in the U.S. every year. Although the average age of an HIV-infected person in the U.S. is approximately 30 years, the number of people living with HIV over the age of 50 years is increasing significantly [3]. This reflects the significant decline in HIV-associated mortality as a result of effective HIV treatment.

## ANTIRETROVIRAL THERAPY

Six different classes of antiretroviral agents (ARV) have been approved by the U.S. FDA (Table **1**). They include: nucleoside reverse transcriptase inhibitors (NRTIs), which block the viral RNA to DNA transcription process; non-nucleoside reverse transcriptase inhibitors (NNRTIs), which inhibit the reverse transcriptase enzyme**;** protease inhibitors (PIs), which inhibit the viral protease enzyme; fusion inhibitors which block the fusion of HIV with the host cell at the initial point of contact; entry inhibitors which block the entry of HIV into the host cell by blocking the cell-surface co-receptor CCR5; and integrase inhibitors which prevent the integration of the viral DNA into the host genome.

Several national and international expert panels have established numerous HIV treatment guidelines. Recommended regimens usually include three different ARV agents in combination, usually from two classes such as 1 NNRTI + 2 NRTIs or 1 PI + 2 NRTIs [4]. Alternate regimens are also provided in the guidelines for select cases where certain ARV drug classes are contraindicated because of drug interactions, ARV resistance, or severe intolerability to preferred regimens. Ritonavir deserves special mention. Because it can inhibit certain liver cytochrome enzymes and therefore the metabolism of certain PIs, it is now used almost exclusively in small doses to increase the blood levels of other PIs, functioning as a boosting agent. A decision to start treatment is based primarily on the CD4 count level and HIV viral load, and secondarily on the readiness of the patient to accept and adhere to life long treatment. In general, treatment is recommended for those patients with a CD4 count < 350/mm^3^. HIV treatment can fail due to the acquisition of resistant virus by transmission from an exogenous source, or development of ARV resistant virus due to poor adherence or subtherapeutic ARV levels secondary to drug-drug interactions. Although multiple ARVs are available for second line regimens, multidrug resistance can be difficult to treat. The goal of HIV treatment is always to achieve an undetectable HIV viral load (< 50 copies/mL). However, in some patients this may no longer be possible due to acquired ARV drug resistance after several failed regimens. There are no formal guidelines on what specific ARVs are to be used in such situations. Interpretation of genotypic or phenotypic resistance tests and prior ARV history require an experienced HIV expert for optimal management.

## ANTIRETROVIRAL MEDICATION SIDE EFFECTS

ARVs in general have become easier for patients to tolerate, but a number of symptomatic side effects are still experienced. The most frequent of these is gastrointestinal intolerance, including nausea, vomiting, and diarrhea. These symptoms are commonly seen with lopinavir/ritonavir, ritonavir in combination with any PI, tenofovir, zidovudine, didanosine, abacavir and raltegravir. Efavirenz is associated with central nervous system effects, including vivid dreams, hallucinations, insomnia, and somnolence. Some of the older NRTIs, such as didanosine and stavudine, are associated with peripheral neuropathy.

Atazanavir is associated with asymptomatic hyperbilirubinemia, while nevirapine may cause fatal hepatic necrosis. Maraviroc, tipranavir, and darunavir can all cause hepatotoxicity, particularly in those patients with hepatitis C virus (HCV) or hepatitis B virus (HBV) co-infection. Pancreatitis is seen most often with stavudine and didanosine. A life threatening lactic acidosis can be seen with any NRTI, but most commonly with stavudine. Stevens-Johnson syndrome can be caused by nevirapine, and abacavir can cause a fatal hypersensitivity reaction characterized by fever, rash, malaise, fatigue, anorexia, and shortness of breath. Zidovudine may cause bone marrow suppression, and tenofovir frequently causes renal dysfunction, but can cause acute renal failure as well. The PIs are associated with osteonecrosis, and a resultant increase in hip fractures has been reported [5].

Many ARVs cause hyperlipidemia or the lipodystrophy syndrome, which is manifested by peripheral fat wasting (lipoatrophy) and visceral fat accumulation (lipohypertrophy) (Table **2**) [6, 7]. This is a class effect of the PIs, and also occurs frequently with the NRTIs, in particular stavudine [8]. The NRTIs are also associated with hepatic steatosis related to mitochondrial toxicity (dysfunction and reduction of mitochondria), most commonly with stavudine and didanosine. Of the NRTIs, stavudine mainly causes increased triglycerides (TG), but may also increase low density lipoprotein cholesterol (LDL-C) and total cholesterol (TC). Of the NNRTIs, efavirenz is more likely to cause hyperlipidemia than nevirapine. All of the PIs except atazanavir have been associated with hyperlipidemia. PIs, and in particular ritonavir-boosted PIs, cause elevations of LDL, TC and TGs and a decrease in high density lipoprotein cholesterol (HDL-C) [9]. PIs suppress the breakdown of transcription factors such as nuclear sterol regulatory element binding proteins (nSREBPs), which are important in lipid homeostasis [10-12]. This leads to accumulation of nSREBP in the liver, causing increased cholesterol and fatty acid synthesis, and reduced triglyceride storage, leading to increased circulating lipids, and in the tissues, causing lipodystrophy and insulin resistance. PIs also inhibit the breakdown of apolipoprotein B *via* binding to a region homologous to HIV-1 protease, thereby causing excess production and circulation of triglyceride-rich lipoproteins [11]. The inhibition of cytochrome P450, in particular by ritonavir, may also disrupt fat metabolism by inhibiting the conversion of retinoic acid to cis-9-retinoic acid, which results in reduced stimulation of the retinoic X receptor (RXR). This in turn prevents adipocyte differentiation and increases adipocyte apoptosis, leading to hyperlipidemia [13]. PIs may also bind to HIV-1 protease homologous regions on two proteins important for lipid metabolism, cytoplasmic retinoic-acid binding protein type 1 (CRABP-1) and low-density lipoprotein-receptor-related protein (LRP) [14]. CRABP-1 prevents binding with retinoic acid and RXR action just as cytochrome P450 does, resulting in hyperlipidemia [13]. LRP is responsible for chylomicron clearance in the liver, and for breakdown of triglycerides in the circulation. Therefore PI binding to LRP also contributes to hyperlipidemia [13].

The metabolic side effects of ARVs have been some of the most difficult to manage. The metabolic syndrome, comprised of abdominal obesity, hyperglycemia, dyslipidemia, and hypertension, is associated with PI therapy. The National Cholesterol Education Program Adult Treatment Panel (NCEP ATP) III definition of metabolic syndrome is at least 3 of the following risk factors: waist circumference >88 cm [women] or >102 cm [men]; blood pressure >130/85 mm Hg or drug treatment for hypertension; triglycerides > 150 mg/ dL; fasting glucose >100 mg/dL; and HDL cholesterol  <50 mg/dL in women or  <40 mg/dL in men [15]. The INITIO study followed patients on NNRTI or PI-based therapy [16]. Of 881 patients in the study, 234 (or 12 per 100 patients-years) developed the metabolic syndrome according to NCEP-III criteria during 3 years of follow-up. For patients who developed the metabolic syndrome during therapy, the hazard ratio of developing cardiovascular disease was 2.73, and the hazard ratio for developing type 2 diabetes mellitus was 4.89. Those patients assigned to PI therapy were more likely to develop the metabolic syndrome on multivariate analysis. Another study found that the incidence of metabolic syndrome was 14% in HIV-infected adults, and was associated with increased levels of leptin and C-reactive protein (CRP), and decreased levels of adiponectin [17].

Type II diabetes has an incidence of 4.4 cases per 1000 person-years of follow-up in HIV-infected patients in the Swiss HIV Cohort [18]. In the Data Collection on Adverse Events of Anti-HIV Drugs (D:A:D) study, DM incidence increased with cumulative exposure to ARVs, and was significantly associated with exposure to stavudine, lamivudine, and didanosine [19]. In a group of minority patients in Los Angeles taking PIs, the incidence of diabetes was 7.2% in 3 years of follow-up [20]. *In vitro* studies demonstrated that the PI indinavir inhibits the activity of GLUT-4, an insulin-sensitive glucose transporter responsible for glucose uptake into muscle and fat cells, thereby contributing to insulin resistance [21, 22]. One PI, atazanavir, did not inhibit GLUT-4 *in vitro*, which helps to explain some of the clinical data showing less insulin resistance in patients taking atazanavir compared to other PIs. Healthy volunteers taking atazanavir have normal rates of glucose disposal, while those taking lopinavir/ritonavir have decreased glucose disposal [23]. Insulin sensitivity was unchanged in healthy volunteers taking atazanavir/ritonavir 300/100 mg once daily, but was significantly decreased in volunteers taking lopinavir/ritonavir 400/100 mg twice daily [24]. Increasing length of exposure to NRTIs is associated with insulin resistance as well. In the Multicenter AIDS Cohort Study, cumulative exposure to NRTIs was associated with fasting hyperinsulinemia while PIs and NNRTIs, surprisingly, were not [25]. Of all the ARVs, stavudine was associated with the highest risk of hyperinsulinemia, and in another study of healthy volunteers, stavudine administration for one month significantly reduced insulin sensitivity [26].

## DRUG INTERACTION ISSUES IN HIV MANAGEMENT

In general, NNRTIs and PIs are metabolized in the liver and can inhibit or induce hepatic P450 cytochrome enzymes. In particular, CYP3A4, 2D6, 2C9, 2B6, and 2C19, could be induced or inhibited by various ARVs. Ritonavir is generally a potent inhibitor of 3A4 and 2D6, but can induce 2C9. Other PIs or NNRTIs could have opposite effects. Thus, metabolism of a number of non-HIV related medications can be affected by, or directly affect HIV medications through drug-drug interactions leading to either significant toxicity or decreased efficacy of the target medications. Because of similar metabolic pathways several CV drugs are contraindicated or must be used with caution in patients receiving certain HIV medications (Table **3**). The statins simvastatin, and to a lesser extent lovastatin, are metabolized *via* CYP3A4. Fluvastatin is metabolized *via* CYP2C9. Rosuvastatin is minimally metabolized *via* CYP2C9 and CYP2C19, and is largely excreted in the stool non-metabolized. Pravastatin undergoes glucuronidation in the liver, and is renally excreted non-metabolized. Protease inhibitors and the NNRTI delavirdine inhibit CYP3A4, and therefore significantly increase levels of simvastatin and lovastatin [27]. NRTIs are not known to interact with statins. Because the NNRTIs nevirapine and efavirenz both induce CYP3A4, they decrease the AUC of statins. Fibrates do not have significant interactions with ARVs. Other drugs such as bepridil, flecainide, propafenone, quinidine, and amiodarone should not be used with certain ARVs because of common metabolic pathways.

Hepatic metabolism of other drugs such as warfarin, calcium channel blockers, and phosphodiesterase type 5 (PDE5) inhibitors (particularly in combination with nitrates) may be inhibited to a lesser degree and should be used with caution. Potential drug interactions exist between calcium channel blockers, in particular amlodipine, nifedipine, and verapamil, with PIs and NNRTIs, where levels of calcium channel blockers may be significantly increased. Thus, the dose of the calcium channel blockers may need to be reduced [29]. Carvedilol interacts with both PIs and NNRTIs, and caution should be used when co-administering it with these drugs [29]. Beta blockers should be used with caution with atazanavir due to the possibility of additive prolongation of the PR interval [29]. It is safe to use atenolol with atazanavir in the absence of ritonavir. No PR prolongation was found in a clinical study of atazanavir, but the QRS interval was increased an average of 5 ms [30]. There are no specific contraindicated drug-drug interactions between antidiabetic medications and antiretrovirals, however additional monitoring may be necessary due to overlapping medication toxicities (e.g., thiazolidinediones/PIs and liver toxicity, metformin/NRTIs and lactic acidosis). Providers should also watch for potential drug-drug interactions between thiazolidinediones and NNRTIs or protease inhibitors, which could result in increased effects of the hypoglycemic agent. Adjust dosages of these antidiabetic drugs as needed. Some PIs (ritonavir, nelfinavir) can increase glucuronidation of fibrates, thus decreasing their effectiveness. Therefore, cardiologists should always look at metabolic pathways when prescribing new medications, particularly to those HIV infected patients who are already receiving ARVs.

In addition to general metabolic alterations, individual patient variation as a result of polymorphic differences in a variety of genes associated with absorption, distribution, metabolism, and excretion (ADME) of medications has been described. For example, alterations in CYP2B6 (AA 516 T/T genotype) have resulted in increased efavirenz plasma concentrations compared to those without this genotype. The HLA-B5701 haplotype has been associated with abacavir hypersensitivity reactions. And active drug transporters such as P-glycoprotein (MDR1) polymorphisms, have also been associated with altered drug transport [31]. Finally, polymorphisms of ABCA1, APOA5, APOC3, APOE, and CETP have been associated with increased lipid levels in HIV infected patients using ARVs [32].

## PATHOPHYSIOLOGY OF HIV-ASSOCIATED CARDIOVASCULAR DISEASE

Chronic inflammation, hypercoagulability and platelet activation all contribute to endothelial dysfunction, and are a probable link between HIV and cardiovascular disease. HIV influences endothelial function *via* activated monocytes and resultant cytokine secretion, and *via* a direct effect of the secreted HIV proteins tat and gp120. A simple measure of the chronic inflammation in HIV-infected patients is the higher levels of high sensitivity C-reactive protein (CRP) found in HIV-infected patients compared to control subjects, indicating a higher risk for cardiovascular events [33]. Other inflammatory markers such as interleukins are also elevated in HIV infection. Endothelial markers are increased as well, including soluble vascular cell adhesion molecule (sVCAM-1), soluble intercellular adhesion molecule (sICAM-1), and von Willebrand factor (vWF) [34]. These markers indicate chronic endothelial activation and subsequent endothelial dysfunction, which may trigger inflammation and a hypercoagulable state. Endothelial activation also triggers platelet activation, which can upregulate adhesion molecules.

Much is known about the direct effects of HIV on endothelial function. For example, it is known that nitric oxide (NO) is a mediator of endothelial dysfunction in HIV infection. NO is produced in excess because of reduced expression of endothelial NO synthase (eNOS) and the resultant increased expression of an inducible NO synthase (iNOS) [35]. Then excess NO reacts with oxygen radicals to produce peroxynitrite, which then causes oxidative damage to the vascular endothelium, and decreased flow mediated dilation [36]. The HIV transactivator of viral replication (tat) protein has been found to significantly decrease eNOS mRNA and thus impair vasorelaxation in porcine coronary arteries [37]. The HIV surface protein gp120 (envelope glycoprotein) also stimulates NO production in activated macrophages, and directly activates endothelial cells resulting in increased adhesion of leukocytes to the endothelium, causing endothelial damage [38, 39]. Clinical evidence of a link between NO and cardiovascular disease was found in a study of HIV-associated cardiomyopathy. Levels of TNF-α and iNOS were significantly higher in HIV-positive individuals with cardiomyopathy on endomyocardial biopsy than in HIV-negative cardiomyopathy patients [40].

The HIV tat protein activates mononuclear cells to secrete cytokines IL-6, IL-8, and TNF-α, which activate endothelial cells and enhances leukocyte adhesion [39]. Both IL-6 and IL-8 are increased in HIV-infected patients and increased levels are correlated with HIV viral load as well as Von Willebrand Factor (vWF), and tissue-type plasminogen activator [41]. HIV tat protein further activates endothelial cells by increasing expression of adhesion molecules that enhance leukocyte adhesion to endothelium [39]. ICAM and VCAM are induced by tat in vascular endothelial cells *in vitro* [42]. ICAM and VCAM levels are increased in HIV-infected patients, and decrease with ARV treatment [34].

Other effects of HIV infection include increases in IFN-γ, which has been associated with hypertriglyceridemia [9, 43]. HIV has also been found to lower HDL-C and increase TG; and during progression to AIDS, LDL-C decreases [44]. Adhesion molecules such as E-selectin and Von Willebrand Factor, which promote the endothelial adhesion of platelets, are also increased in HIV infection [39]. Platelet activation is increased *via* vWF in HIV infection, contributing to an increased incidence of thromboembolic events [45]. Activated coagulation factors and reduced anticoagulants also are important in contributing to thromboembolic events in HIV infection. Activation of coagulation mechanisms may occur through endothelial activation and inflammation [34]. The HIV envelope protein gp120 has been shown *in vitro* to activate smooth muscle cells *via* the chemokine receptors CXCR4 and CCR5, and thus to cause them to express tissue factor [46]. Tissue factor, in turn, initiates the coagulation cascade leading to thrombosis. Coagulation abnormalities in HIV include decreases of protein S levels by 73%; increased levels of anticardiolipin antibodies and lupus anticoagulant, and decreased levels of antithrombin are also seen [39, 47].

Finally, HIV-induced apoptosis is another mechanism of endothelial injury. HIV Gp120-induced apoptosis has been implicated in lung endothelial injury contributing to pulmonary hypertension, and HIV tat has been associated with endothelial apoptosis *in vitro* [39]. Taken together, it is clear that HIV abnormally affects many aspects of cardiovascular physiology.

Many studies have evaluated the effect of HIV on endothelial function, using endothelial function as a proxy for cardiovascular risk. Endothelium-dependent vasodilatation, as measured by flow-mediated dilation (FMD) of the brachial artery, was significantly diminished in 75 HIV infected patients compared to HIV-uninfected controls in a multivariate analysis study [35, 48]. In this study, smoking, male sex, and obesity were also independently linked to decreased FMD, which is a reminder of the association of smoking and obesity with endothelial dysfunction, and the importance of addressing these risk factors in HIV-infected patients. PI therapy was not an independent risk factor for endothelial dysfunction. In a separate study, 22 HIV-infected patients taking PIs did have impaired FMD compared to 15 HIV-infected patients not taking PI therapy. The group taking PIs had significantly higher levels of chylomicron, VLDL, LDL, and HDL cholesterol levels, which likely contributed to the endothelial dysfunction seen [49]. A study in rats examined whether the NRTI zidovudine (AZT) causes endothelial dysfunction. AZT alone, and AZT plus indinavir both decreased vessel relaxation [50]. Therefore NRTIs and PIs may both play a role in the endothelial dysfunction seen in HIV-infected patients. However, since ARVs reduce the endothelial activation caused by uncontrolled HIV infection, their overall effect on endothelial function is still an area of active research and controversy [51].

## CARDIOVASCULAR EPIDEMIOLOGY OF HIV INFECTION

Premature coronary artery pathology has been reported among HIV-positive individuals. Autopsy studies first suggested an association between vascular endothelial pathology and HIV [52]. Reporting of clinically evident coronary artery disease (CAD) has increased since the introduction of PIs in 1996. HIV infected patients have higher rates of MI than non-HIV infected patients, 11.13 versus 6.98 per 1000 person-years in a recent US study [53]. A recent Danish study found that HIV infected patients receiving highly active antiretroviral therapy (HAART) were more likely to be hospitalized with ischemic heart disease than HIV-uninfected controls [54]. Similarly, a Kaiser Permanente study found a higher rate of cardiovascular events in HIV infected patients who were not receiving ARV therapy compared to HIV-uninfected controls [55].

Several large studies have compared rates of CAD among HIV-positive patients who are taking PIs compared to those who are not. The HIV Outpatient Study (HOPS) found increased frequency of MIs after the introduction of PIs (p=0.0125), and a hazard ratio of 7.13 by univariate analysis for myocardial infarction in those who used PIs [56]. The hazard ratio on multivariate analysis was 6.51 but did not reach significance. Corroborating this finding was an Italian study that found patients who received PIs had an annual coronary event rate of 5.1/1000 while those who received NNRTIs had an event rate of 0.4/1000 (P<0.001)[57]. Another study using data from the HOPS study and other outpatient clinics found event rates of 9.8/1000 and 6.5/1000 for patients who had and had not been exposed to PIs, respectively (p=0.0008) [58]. A study of the French Hospital Database found an increase in the relative risk of MI with increasing length of time on PI therapy [59]. In a follow-up study combining data from HOPS and the Athena national cohort in the Netherlands, an increased risk of MI was found in those on PI therapy (hazard ratio 1.19, P= 0.04), but not on NNRTI therapy [60]. However, a reduction in a combined endpoint of death and atherosclerotic events of patients on NNRTI or PI-based therapy compared to patients not on therapy was found, underscoring the importance and positive benefits of HAART.

The D:A:D study also found that the incidence of MI increased with length of time on HAART, with an adjusted relative risk of 1.26 (26%) per year of exposure, though the absolute incidence of cardiac events was low at 3.5 per 1000 person-years [61]. A subsequent analysis of the D:A:D study separating the effect of PIs versus NNRTIs found that patients taking PIs had an increased relative risk of MI of 16% per year, while the risk of MI for patients taking NNRTIs was not significantly increased [62, 63]. When adjusted for lipid levels, diabetes mellitus, and hypertension, the relative risk of MI per year was increased only 10%, suggesting that lipid changes accounted for some, but not all, of the risk associated with PI therapy. Another recent finding from the D:A:D study is that abacavir and didanosine were independent risk factors for MI. Recent use of abacavir or didanosine, regardless of duration of use, was associated with a 90% and 49% increased risk of MI, respectively. Zidovudine, stavudine, and lamivudine were not associated with increased risk of MI [64].

However, it is important to remember that the increase in absolute risk attributable to PIs is small. It should be emphasized that the significance of an increased risk of CAD is dependent on the patient’s underlying cardiac risk factors. Since risk of CAD is increased by a factor of 1.16 per year of PI therapy, or a doubling of risk over a 5-year period, PIs will confer minimal increased risk to someone with a low starting risk of CAD [65]. The importance of addressing underlying cardiac risk factors, such as tobacco use, must be emphasized. Some of the effects of PI therapy, such as dyslipidemia and insulin resistance, can be medically managed to further reduce CV event risk.

Other studies have reported a decrease in CV risk in patients receiving ARV therapy. HAART appeared to decrease the risk of cardiac disease in the Strategies for Management of Antiretroviral Therapy (SMART) study. Patients in the ARV-sparing arm had an increased rate of cardiac events compared to those who remained on HAART [66]. The increased rate of cardiac events could be related to the increase in inflammation caused by rising HIV viral loads in patients who stopped HAART. A recent study looked at this possibility, and found significantly increased levels of IL-6 (30% versus 5%) and D-dimer (16% versus 0%) in patients who discontinued HAART compared to those who continued HAART [67]. These increased levels of IL-6 and D-dimer were associated with cardiovascular events. A similar treatment interruption study, the STACCATO trial, found increased sVCAM, adiponectin, and IL-10 in the patients who had a treatment interruption versus those who stayed on ARV therapy [68]. Another treatment interruption study found that increases in viral load correlated with increases in sVCAM [69]. Corroborating the beneficial effects of HAART on cardiovascular risk, a large retrospective U.S. Department of Veterans Affairs (VA) study found that the admission rate for cardiovascular and cerebrovascular disease decreased from 1.7 to 0.9 per 100 patient-years from 1995 to 2001, during the time that HAART with ritonavir-boosted protease inhibitors became widely used. No association was found between cardiovascular events and the use of PIs, NNRTIs, or NRTIs [70]. The mean follow-up time was 15 months, and the mean time on PIs was 16 months, so this study may speak more to the short-term benefits of ARV treatment rather than the long term cardiovascular effects of HAART. Overall, these data on the incidence of CAD and MI in HIV-infected individuals support an initial decrease in cardiac risk with the use of ARV therapy, related to a reduction in inflammation and endothelial dysfunction caused by HIV. They do point to an increased cardiovascular risk in those on PI-based therapy, which increases with time spent on therapy.

HIV and HAART have also been associated with increased carotid artery intima-media thickening, which is used as a surrogate for cardiovascular risk. Baseline intima-media thickness (IMT) is higher in patients with HIV infection than those without, and progresses at a faster rate [33, 71]. Increased IMT is associated with classic cardiovascular risk factors, such as older age, higher LDL cholesterol, smoking pack-years, and hypertension. ARV therapy has also been implicated; Jerico *et al.* found that ARV therapy was associated with subclinical atherosclerosis [72]. Along with PI exposure, lower CD4 count and genetic polymorphisms are also associated with progression of IMT [73, 74]. One of these genetic polymorphisms is in the monocyte chemoattractant protein-1 gene (MCP-1-2518G), and is associated with increased expression. MCP-1 attracts circulating monocytes to endothelial cells where they become foam cells which lodge in the endothelium and contribute to atherosclerosis [74, 75]. Mutations of stromal derived factor-1, (SDF1), which is a chemokine receptor upregulating chemo-attraction and adhesion, were shown to cause platelet activation in atheromas, migration of smooth muscle cells to the endothelium, increased expression of CX3CR1, and were associated with lower IMT increases [74]. Another study that found higher carotid IMT in HIV-infected subjects compared to controls was independently associated with cytomegalovirus (CMV)-specific T-cell responses in the HIV-infected population [76]. Carotid IMT has also been found to be higher in HIV-infected children [77]. Other studies have found no association between HIV infection or PI therapy and IMT or IMT progression [78, 79]. However, the baseline IMT in these studies was much lower than other studies, suggesting a lack of sensitivity in detecting IMT changes [80]. It is most likely that HIV and PI therapy both increase IMT, but that the factors most important in determining atherosclerotic disease are traditional risk factors.

Left ventricular dysfunction, dilated cardiomyopathy, and myocarditis all occur with increased frequency in AIDS patients. In a pre-HAART study, global LV hypokinesis was found in 14.5% of patients and was associated with lower CD4 counts, and congestive heart failure was diagnosed in about 2% of patients [81]. Causes may include a direct effect of HIV, other cardiotropic viruses, ARV toxicity, cytokines, opportunistic infections (OIs), illicit drug use, or nutritional deficiencies. In a pre-HAART series of myocardial biopsies in HIV patients, 5 of 33 stained positive for HIV while 16 of 33 were positive for CMV using antisense riboprobes [82]. The incidence of cardiomyopathy is declining post-HAART [83]. Myocarditis has many causes in HIV-infected patients, though a specific diagnosis is rare. These include toxoplasmosis, tuberculosis, *Cryptococcus neoformans*, Aspergillus, Candida, cytomegalovirus, HSV, *Mycobacterium avium intracellulare*, and HIV [84, 85]. In an Italian study, lymphocytic interstitial myocarditis was documented in 30 of 440 (7%) of AIDS patients at autopsy [86]. Again, the incidence of myocarditis has also declined in the era of effective HIV treatment.

Pericardial disease is common in HIV-infected patients. In the Prospective Evaluation of Cardiac Involvement in AIDS (PRECIA) study, before the introduction of HAART, the incidence of pericardial effusion was 11% per year, and it was associated with shorter survival and lower CD4 counts [87]. A majority of the effusions were asymptomatic and idiopathic. They rarely cause tamponade [85]. Various OIs and malignancies have been reported to cause pericardial effusions, including Kaposi’s sarcoma, mycobacteria, CMV, prosthetic valve endocarditis, bacterial pericarditis caused by organisms such as *Streptococcus pneumoniae* [88], Nocardia [89, 90], lymphoma, and immune reconstitution inflammatory syndrome secondary to *Mycobacterium tuberculosis* (TB) [91]. In Africa, the majority of pericardial disease in HIV infected patients is caused by TB [84, 92, 93]. The incidence of pericarditis has also been decreasing since the introduction of HAART. In a retrospective study of the incidence of cardiac disease comparing the era of mono or dual NRTI therapy versus HAART, the incidence of pericarditis had decreased from 13.5% to 3.4% [83]. Bundle branch block, atrial fibrillation, ischemia, and dilated cardiomyopathy have similarly decreased. It is unknown if the incidence of pericardial effusion has declined in the HAART era. However, the direct effects of HIV and OIs on cardiovascular tissue and the associated cardiac disease have been significantly reduced as a result of effective HIV treatment.

Thromboembolic disease has a high prevalence in HIV-infected patients, including deep vein thrombosis, pulmonary embolism, thrombotic microangiopathy, and retinal venous thrombosis [39]. A retrospective review found a rate of deep venous thrombosis ten times that of HIV-uninfected patients in the general population [94]. Thromboembolic events have been found to occur at higher rates in patients with a CD4 count <200/mm^3^ compared to those with a CD4 >200/mm^3^ [95]. Fultz at all looked a 37,535 HIV-infected veterans and the same number of controls, and found an increased rate of venous thromboembolism in the HIV-infected veterans both before 1996, in the pre-HAART era, and after 1996, in the HAART era after protease inhibitors became available for treatment [96]. A study by Lijfering *et al.* found that patients with CD4 <200/mm^3^ had higher factor VIII and fibrinogen concentrations and lower protein S concentrations, perhaps explaining some of the difference in thrombosis rates [97]. Other factors underlying thromboembolic events in HIV-infected patients are discussed in a previous section of this paper.

Pulmonary hypertension is significantly increased in HIV infected patients compared to the general population, with an incidence of around 0.5% reported in 1991 [98]. The same incidence (0.46%) was found in a 2008 study, thus refuting the hypothesis that the incidence had decreased since the introduction of HAART [99, 100]. Though HAART improved mortality in HIV-infected patients with pulmonary hypertension in a prospective study of the Swiss HIV Cohort, the median survival was poor at 2.7 years from diagnosis [101]. Therefore, pulmonary hypertension portends a poorer prognosis in HIV-infected patients, despite HAART therapy [102].

The rates of endocarditis are similar among HIV-infected patients with shared risk factors, such as injection drug use, and non-HIV-infected individuals. HIV per se does not seem to be a risk factor for endocarditis, though mortality may be higher for those with CD4 counts under 200/mm^3^ [85, 103].

Finally, cardiac neoplasms are a rare complication of AIDS. B cell lymphomas can be primary cardiac lymphomas or part of disseminated disease. Kaposi’s sarcoma can rarely invade the heart in disseminated disease. Both of these neoplasms are more common in HIV-infected than in HIV-uninfected patients [85].

## PRIMARY CARE AND CO-MORBID CONDITIONS ASSOCIATED WITH ARVS

Because of the high rate of morbidity and mortality associated with cardiovascular disease in HIV-infected patients, it is important to identify those at risk so they can be targeted for aggressive risk-management. Risk factors for cardiac disease include increased age, male sex, smoking, elevated LDL-C, low HDL-C, hypertension, and the presence of diabetes or peripheral vascular disease [104]. HIV-infected men have a greater number of these traditional CAD risk factors compared to HIV-uninfected controls [105]. The Framingham Risk Score (FRS) has been validated as a way to quantify 10-year coronary heard disease risk based upon age, sex, total cholesterol (TC), HDL-C, smoking status, and systolic blood pressure in the general population [106]. A link to the 10-year risk calculator can be found at http://www.nhlbi.nih. gov/guidelines/cholesterol. When applied to the HIV-infected population in the D:A:D study, the FRS slightly underestimated the number of CHD events for patients on ARV therapy, but in ARV-naïve patients, it overestimated the risk [107]. This may be for reasons including the direct effect of HIV on the endothelium, an effect of PI therapy apart from its metabolic effects (*via* up-regulation of CD36, which is a receptor on macrophages increasing cholesterol uptake and promoting foam cell formation and atherosclerosis [108]), an increased differential effect of cardiac disease risk factors on HIV patients, or the fact that in general, we are applying the Framingham study to a younger HIV infected population than the one in which it was developed [109]. Since the FRS estimates 10-year cardiovascular risk, it may be too short to represent the lifetime risk of younger or middle-aged HIV-infected patients [110]. A new prediction tool developed from the D:A:D study, which also includes family history, the TC/HDL-C ratio, the presence of diabetes, and the duration of exposure to PIs, performed better than the FRS in predicting CHD in this cohort [109, 111].

Given the higher prevalence of subclinical atherosclerosis in HIV-positive individuals discussed above, carotid ultrasonography can be considered as a way to assess cardiovascular risk in this population. Maggi *et al.* found that carotid vascular wall lesions were associated with PI therapy, and recommended screening ultrasonography for this group to guide cardiovascular risk management [112]. Another means of assessing cardiovascular risk is the high-sensitivity C-reactive protein (CRP). This has been associated with traditional cardiovascular risk factors in HIV-infected patients, including waist-to-hip ratio, fasting glucose, NNRTI therapy, PI therapy, and the metabolic syndrome [113]. Elevated CRP is a marker of visceral fat accumulation and predicts higher cardiovascular mortality in HIV-infected women [114]. Reingold *et al.* found that male patients with HIV monoinfection had an 88% higher CRP level than controls, while there was no difference in women [114]. However, HCV co-infection was associated with 50% lower CRP levels. CRP levels were strongly correlated with visceral adipose tissue and subcutaneous adipose tissue. Of interest is that CRP levels did not correlate with CD4 count or HIV viral load, or with ARV therapy, though other studies have found an association between CRP and ARV therapy [115]. In this study, by Masiá *et al.*, CRP elevation of 0.3 mg/dL or greater occurred in fully 40% of HIV-infected patients studied. CRP levels can help stratify cardiovascular risk in moderate-risk patients in the general population, and may have a role to play in risk stratification in HIV-infected patients as well.

Smoking has been identified as the most important modifiable CAD risk factor in HIV-infected patients [116]. In the SIMONE study, a national Italian study of HIV-infected patients, smoking was the main contributor to coronary heart disease (CHD) risk in HIV-infected patients [117]. HIV-infected patients have been found to have high rates of smoking in several studies, including in a combined analysis of the MACS study and the Women’s Interagency HIV Study (WIHS), which found about 35-40% of HIV-infected patients were smokers [105]. Among men in this study, HIV-infected patients had higher smoking rates than in HIV-negative controls. In the D:A:D study, more than 50% of the patients were former or current smokers, and they had double the risk of MI [118]. In the SIMONE study, the smoking prevalence was 60% [119]. In the Swiss HIV Cohort the prevalence of current smokers was 47.6% in 2006 [120]. A discussion of smoking cessation methods is beyond the scope of this paper, but various programs have been found to be effective in the HIV-infected population [121, 122].

Diabetes is also an important risk factor for CAD; like smoking, it has a higher prevalence in HIV-infected patients than in the general population. In the Multicenter AIDS Cohort (MACS) study, the incidence of diabetes in HIV-infected men on HAART was more than four times that of HIV-seronegative men [123]. The metabolic syndrome has a similar prevalence of approximately 25% among HIV-infected and HIV-uninfected controls, but a higher proportion of HIV-infected patients with the metabolic syndrome have diabetes [124]. Lifestyle therapy is important in the treatment of metabolic syndrome and diabetes. The Diabetes Prevention Program found that patients with impaired glucose tolerance who underwent a diet and exercise program were 41% less likely to develop the metabolic syndrome [125]. Though this study did not specifically study HIV-positive patients, the results should be applied to all patients. Glycemic goals, according to the American Diabetes Association (ADA) are a HbA1C of ≤ 7% for most patients, with an option of tighter control to 6% for selected patients [126].

As well as addressing cardiac risk factors in diabetics, diabetics with HIV infection who are over age 40 or have any cardiac risk factors should all receive low-dose aspirin therapy in accordance with ADA recommendations. There is no known contraindication to ASA therapy in HIV-positive individuals. In a study of the management of diabetes in HIV-infected individuals, only 5% were receiving the recommended ASA therapy [127]. Along the same lines, according to the American Heart Association, aspirin therapy should be recommended for any patient with an FRS of 10% or greater as primary prevention of MI and stroke, and the data from the D:A:D study, among others, suggests that HIV-infected patients have high levels of cardiac risk and disease, and should have the same therapeutic interventions to prevent CAD as HIV-uninfected patients [128].

Hypertension in HIV-infected individuals should be treated according to JNC 7 guidelines [129]. Updated hypertension guidelines (JNC 8) are scheduled to be released in 2009. Anyone with a blood pressure reading of >140/90 mmHg on two readings on separate days should be treated for hypertension. Those with diabetes, chronic kidney disease, proteinuria, CAD equivalent, or a FRS ≥ 10% should be treated with a goal blood pressure of <130/80 mmHg. Lifestyle modifications are recommended for any HIV-infected patient with hypertension, including weight loss if appropriate, sodium restriction and healthy diet, exercise, smoking cessation, and moderation in alcohol consumption. If systolic blood pressure is ≥160 mm Hg, or diastolic blood pressure is ≥100 mm Hg, then treatment should be started with 2 drugs simultaneously. Thiazide diuretics are first-line therapy unless there is a compelling indication for alternative treatment; such as beta-blockers (use with caution with atazanavir) or angiotensin-converting enzyme (ACE) inhibitors for heart failure; beta-blockers or calcium-channel blockers (start with low doses of amlodipine, nifedipine, and verapamil in HIV-infected patients) for ischemic heart disease; ACE inhibitors or angiotensin receptor blockers (ARBs) for chronic kidney disease and diabetes; and ARBs or ACE inhibitors for left ventricular hypertrophy. Hypertension management is an important component of lowering overall risk of cardiovascular disease including stroke and MI in HIV-infected patients.

Lifestyle changes are the first line of therapy for ARV-associated dyslipidemia and metabolic syndrome. A 6-month intensive lifestyle modification program was able to significantly reduce waist circumference, systolic blood pressure, hemoglobin A1C, and lipodystrophy, but not serum lipid levels, in HIV-infected patients with the metabolic syndrome [130]. Dietary and exercise interventions were successful in lowering cholesterol levels 11% in one study [131]. Diet and exercise reduced TC by 18% and TGs by 25% in another study [132]. In a comparison of fish oil supplementation versus diet and exercise, triglyceride levels were decreased 19.5% in the fish oil plus diet and exercise group versus only 5.7% in the diet and exercise group [133].

Guidelines for the management of CHD risk factors in HIV-infected patients were published in 2003, and do not differ significantly from those for HIV-uninfected patients, and are based on the Adult Treatment Panel III (ATP III) guidelines from 2001 [9]. An update of the ATP III guidelines was published in 2004, and the ATP IV guidelines are expected to be released in 2009 [134]. According to the ATP III guidelines and update, a fasting lipid profile should be done on all patients before initiating ARVs, and 3-6 months after starting a new ARV regimen. It can then be done yearly if no lipid abnormalities are detected. If a patient has triglyceride levels greater than 200 mg/dL prior to initiating ARV therapy, a lipid panel should be done within 1 to 2 months after starting ARV therapy, as TGs greater than 500 mg/dL put patients at increased risk of pancreatitis and require prompt treatment. LDL-C is the primary target for lipid-lowering therapy, unless TGs are found to be >500 mg/dL, in which case therapy with a fibrate should be started preferentially.

The lipid goals are based upon Framingham risk factors, and in general should be applied equally to HIV-infected and HIV-uninfected patients. Patients considered high risk are those with a CHD risk equivalent including a previous cardiovascular event, diabetes, cerebrovascular disease or peripheral vascular disease, or, two or more cardiac risk factors (total cholesterol ≥240 mg/dL, systolic blood pressure ≥140 mmHg, diastolic blood pressure ≥90 mmHg, and smoking) with a 10-year calculated CHD risk of >20%. In high risk patients, the treatment goal is an LDL-C less than 100 mg/dL. If the LDL-C is >100 mg/dL, lifestyle changes should be initiated and drug therapy considered. If the LDL-C is already less than 100 mg/dL, drug therapy with an LDL-C lowering drug should also be considered. However, if the patient is very high-risk, including those with acute coronary syndrome, or known cardiovascular disease combined with diabetes, metabolic syndrome, poorly controlled risk factors, or ongoing tobacco use, the LDL-C goal may be set to less than 70 mg/dL. If a patient is at moderately high risk, with ≥ 2 risk factors, and a calculated risk between 10-20%, the LDL-C treatment goal is < 130 mg/dL, with initiation of drug therapy if the LDL-C is > 130 mg /dL. For moderately high risk patients, there is an option to set the treatment goal at an LDL-C < 100 mg/dL, and to use drug therapy if the LDL-C is between 100 and 129 mg/dL. Another metric for LDL-C goals for high and moderately high risk patients is to reduce the LDL-C by 30%, regardless of starting LDL-C level. For those patients with a moderate risk (FRS is <10% and 2+ risk factors), drug therapy should be considered at an LDL-C of 160 mg/dL. If the patient only has 0-1 risk factors then drug therapy would not be recommended until the LDL-C level reaches 190 mg/dL [134]. No specific goal for HDL-C has been set, though some trials have found treating patients with low HDL-C and high triglycerides reduces the risk of CHD. Nicotinic acid has been found to raise HDL-C and lower CHD risk. Another lipid target set forth for patients with TGs>200 is non-HDL-C, which is a measure of VLDL and LDL-C. This target is 30 mg/dL higher than the LDL-C goal. It takes into account remnant lipoproteins in patients with high TGs [134].

Consideration should also be given to changing antiretroviral agents prior to the initiation of lipid-lowering medications. The benefits of switching ARV regimens are proven but appear to be modest compared to the use of lipid-lowering agents, probably because all ARV regimens affect lipid metabolism to some degree. A one year study of HIV-infected adults on HAART including a PI with mixed hyperlipidemia of TG >200 mg/dL and TC >250 mg/dL compared switching the PI to an NNRTI versus adding a statin [135]. Initiation of pravastatin or bezafibrate was more effective than switching to nevirapine or efavirenz in lowering TC, LDL-C, and TGs, and in raising HDL-C. The percentage of patients who reached normal cholesterol levels was 56% in the pravastatin arm, 42% in the bezafibrate arm, 24% in the nevirapine arm, and 12% in the efavirenz arm. Therefore lipid management was more effective after adding lipid-lowering therapy than switching ARV regimens. In the Switch to Another Protease Inhibitor (SWAN) study, patients on ritonavir boosted or unboosted PIs switched to unboosted atazanavir, or ritonavir boosted atazanavir if receiving tenofovir [136]. Switching therapy to atazanavir was successful in lowering TC and TG. Virologic rebound was lower in patients switched to an atazanavir-containing regimen (7% versus 16% kept on their old regimen), confirming that it does not put suppression of HIV viral replication in jeopardy to do so. In general, if a patient has sustained viral suppression, then switching ARV therapies to reduce lipid levels is safe [137]. Switching ARVs has also improved insulin resistance. Switching from indinavir/ritonavir or lopinavir/ritonavir to atazanavir/ritonavir improved insulin resistance in all 9 HIV-infected men evaluated in one study [138].

For patients with elevated LDL-C or with elevated non-HDL-C levels along with TG levels that are 200-500 mg/dL, therapy with a statin is recommended as first line treatment [9]. Fibrates are recommended if the serum TGs are >500 mg/dL. The statins with the lowest potential for interaction with antiretroviral therapy are pravastatin, at a starting dose of 20-40 mg daily, and fluvastatin, starting at 20 mg daily. Low-dose atorvastatin (10 mg) is also a safe starting dose. Pravastatin cannot be used in combination with darunavir, because darunavir increases the AUC of pravastatin by 81% [139]. Low-dose atorvastatin (10mg daily), or rosuvastatin (10-40 mg daily), though both acceptable, may require closer monitoring because of increased drug interactions. Rosuvastatin at 10 mg daily was effective in reducing total cholesterol and triglycerides in HIV-infected patients with PI-induced hyperlipidemia [140]. However, rosuvastatin should be used with caution as the AUC and Cmax are increased 2.1 to 4.7-fold in combination with lopinavir/ritonavir in healthy volunteers [141]. For all patients on PIs, statins should be started at the lowest dose, and increased as needed according to the therapeutic response and toxicity monitoring. Simvastatin and lovastatin are contraindicated in patients taking PIs because blood levels are increased significantly by PIs [27], and concomitant use has been associated with rhabdomyolysis [142-147]. All statins are safe with the NRTIs and NNRTIs, though statin blood levels may be reduced with NNRTIs. The pharmacokinetics of raltegravir with pravastatin is currently being studied, but as raltegravir does not undergo metabolism by the CYP450 system, drug interactions should be minimal. Maraviroc is metabolized through the CYP3A isoform, but does not inhibit the CYP450 system, so would not be expected to raise statin levels, but no data is available [148].

It has been difficult to reach all cholesterol goals in HIV infected patients with statins alone [136, 149, 150]. If further drug therapy is needed, ezetimibe and fibrates are safe with all ARVs, with no and minimal interactions, respectively. Fibric acid derivatives may modestly reduce LDL-C levels in patients with normal TGs, but in patients with elevated TGs, LDL-C levels rise slightly [151]. Three fibrates, bezafibrate, gemfibrozil, and fenofibrate, were compared in a randomized trial for the management of ARV-associated hypertriglyceridemia and hypercholesterolemia [152]. All drugs performed similarly, with reductions of 41% in TGs and 23% in TC. Combination therapy with fenofibrate and pravastatin was also studied in HIV subjects with LDL-C >130 mg/dL and TG >200 mg/dL [151]. Each drug was initiated singly, and then the other was added when lipid level thresholds were not met. Of those on fenofibrate who then added pravastatin, 16% achieved National Cholesterol Education Program III (NCEP III [153]) goals of LDL-C <100 mg/dL and TG <200 mg/dL for patients with two or more cardiac risk factors, while 5% of those who initiated pravastatin first and then added fenofibrate did. This suggests that sequential therapy starting with fenofibrate and then pravastatin is preferable, but few patients reached all target lipid levels with the combination. One retrospective study found the addition of statins and fibrates allowed more HIV patients taking ARVs to reach their cholesterol goals, but the success rates were also relatively low with less than 20% reaching NCEP II goals [150]. Caution must be used in combining fibrates with certain statins because of the increased risk of rhabdomyolysis [11, 147].

Alternative lipid-lowering therapies to statins are an area of active study in HIV. A study of combination fish oil 3 g twice daily and fenofibrate 160 mg once daily for HIV patients not achieving TGs <200 mg/dL with either agent alone found that the combination provided improved TG-lowering effects over either drug alone, achieving the goal in 23% of patients [154]. Ezetimibe has also been studied as an addition to lipid management in HIV-infected individuals. It was given to patients who had an LDL>130 mg/dL despite treatment with pravastatin [155]. At 6 weeks there were significant falls in TC, LDL-C, and TGs, but these benefits diminished by week 24. HDL-C levels increased throughout the study. Ezetimibe monotherapy has also been studied in HIV-infected patients [156]. Patients with an average baseline LDL-C of 121 mg/dL were started on ezetimibe or placebo. LDL-C decreased an average of 12% for patients receiving ezetimibe versus 3% for placebo. There were no significant changes in TGs or HDL-C.

Niacin is not a first-line choice for lipid therapy because of its propensity to cause insulin resistance [9]. However, it was tolerated when used for treatment of low HDL-C levels in HIV-infected subjects, and was associated with a significant decrease in intra-abdominal fat [157]. Bile-sequestering resins should be avoided in HIV-infected individuals because they may be associated with increased triglyceride levels and their effect on antiretroviral absorption is unknown [9].

## INTERVENTIONAL CARDIOLOGY IN HIV INFECTED PATIENTS

Solid organ transplantation has now become a successful reality in HIV infected patients. The first successful heart transplant in an HIV-infected individual was performed in 2001, in a patient with AIDS and a history of multiple opportunistic infections [158]. The patient lived for 3.5 years post-transplant. However, the cause of death was not reported [159]. A second heart transplant was reported in 2003 in a patient with normal CD4 counts and an undetectable HIV viral load [160, 161]. The patient had an uneventful recovery. Another patient who acquired HIV from blood products transfused after her heart transplant has continued to do well with no major complications 10 years after her transplant [162]. A number of successful liver and kidney transplants have been performed in HIV-positive patients (19 and 26, respectively, in one review), with short-term survival rates comparable to non-HIV-infected recipients [163]. Though no statement has been made by national transplant organizations regarding transplant in HIV-positive patients, there is a growing consensus that HIV infection should not be an absolute contraindication to solid organ transplantation. It has been suggested that HIV positive transplant candidates should have undetectable viral loads, normal CD4 counts, and no history of opportunistic infections [160]. However, because opportunistic infections can now be controlled with antimicrobials and immune reconstitution following HIV treatment, this is no longer felt to be an absolute contraindication, and only opportunistic infections with no reliable treatment, such as progressive multifocal leukoencephalopathy, chronic cryptosporidiosis, and drug-resistant fungal infections should be contraindications for transplant [164].

Reports of percutaneous coronary intervention (PCI) have provided mixed results. An observational study of 50 HIV+ and 50 HIV- patients undergoing PCI with the majority receiving stents, and all patients treated with aspirin and clopidogrel at discharge, found no deaths and no difference in restenosis rates (14% v 16%, p = 0.78) at 321 days mean follow-up [165]. However, 5 of 12 HIV infected patients followed after stenting in another study had in-stent restenosis after a mean follow-up of 16 months [166]. Of 24 HIV+ patients admitted with acute myocardial infarction in another study, 20% had reinfarction after discharge, and 43% of those who had PCI, ended up requiring target vessel revascularization [167]. There is a report of late in-stent restenosis in an HIV-positive patient with a drug-eluting stent 23 months after placement, and lifelong aspirin and clopidogrel therapy have been recommended [168].

A study of 27 HIV infected patients undergoing coronary artery bypass graft (CABG) compared to case controls found equal rates of major adverse cardiac events at 30 days, but increased need for revascularization at a mean follow-up of 41 months (42% versus 25%, p=0.03) [169]. Another series of 37 patients looking at outcomes of cardiac surgery including CABG and valvular replacement found acceptable outcomes of freedom from class III angina or heart failure symptoms, MI, death, and repeat revascularization of 81% of patients at 3 years [170]. However, a note of caution was sounded in mentioning 6 needlestick injuries in these 37 surgeries, none of which resulted in HIV seroconversion. In conclusion, it appears that revascularization techniques including PCI and CABG produce good clinical outcomes in HIV+ patients, though a higher need for subsequent revascularization procedures has been documented.

## CONCLUSIONS

It is likely that cardiologists will see HIV infected patients in consultation for medical or interventional management of CV associated disorders. As the HIV population in western countries ages, it is also likely that the prevalence of CV disease in the HIV infected population will increase and cardiologists will have to balance HIV care with management of other co-morbid conditions commonly associated with HIV such as hypertension, diabetes, and hyperlipidemia. Successful management requires understanding HIV specific issues, and coordination of care with the HIV specialist.

## Figures and Tables

**Table 1:** Antiretroviral Agents for Treatment of HIV Infection

Drug Class	Available Agents *generic (common abbreviation) – alphabetic order*
Nucleoside reverse transcriptase inhibitor (NRTI)	Abacavir (ABC) Didanosine (ddI) Emtricitabine (FTC) Lamivudine (3TC) Stavudine (d4T) Tenofovir (TDF) Zidovudine (AZT or ZDV)
Non-nucleoside reverse transcriptase inhibitor (NNRTI)	Delavirdine (DLV) Efavirenz (EFV) Nevirapine (NVP) Etravirine (ETV)
Protease inhibitor (PI)	Atazanavir (ATV) Darunavir (DRV) Fosamprenavir (fAPV) Indinavir (IDV) Nelfinavir (NFV) Ritonavir (RTV) Saquinavir (SQV) Tipranavir (TPV)
Fusion inhibitor	Enfuvirtide (T-20)
Entry inhibitor	Maraviroc (MAC)
Integrase inhibitor	Raltegravir (RAL)
Combination agents – NRTIs Lamivudine/Zidovudine (Combivir®) Abacavir/Lamivudine (Epzicom®) Tenofovir/Emtricitabine (Truvada®) Abacavir/ Lamivudine/Zidovudine (Trizivir®) Combination agents – NNRTI + 2 NRTIs Efavirenz/Emtricitabine/Tenofovir (Atripla®) Combination agents – PIs Lopinavir/Ritonavir (LPV/r) (Kaletra®)	

**Table 2:** Cardiovascular Associated Adverse Effects of HIV Medications

**Cardiovascular Effects**

Antiretroviral: Possibly PIs and other ARVs with unfavorable effects on lipids (e.g. EFV, d4T)
Onset: months to years after beginning of therapy
Presentation: premature coronary artery disease
Prevalence: 3–6 per 1,000 patient-years
Other risk factors for cardiovascular disease such as smoking, age, hyperlipidemia, hypertension, diabetes mellitus, family history of premature coronary artery disease, and personal history of coronary artery disease
Monitoring: identify hyperlipidemia or hyperglycemia, cardiac risk factors
Treatment: Lifestyle modifications: diet, exercise, and/or smoking cessation; switch to agents with less propensity for increasing cardiovascular risk factors, i.e., NNRTI- or ATV-based regimen & avoid d4T use

**Hyperlipidemia**

Antiretroviral: All PIs (except ATV); stavudine (d4T); Efavirenz (EFV) (to a lesser extent)
Onset: weeks to months after beginning of therapy
Presentation: increased LDL & total cholesterol (TC) & triglyceride (TG), decreased HDL Varies with different agents
Prevalence: Increased TC & TG – 1.7–2.3x higher in patients receiving (non-ATV) PI; dependent on underlying hyperlipidemia. Risk based on ARV therapy, PI: LPV/r & RTV -boosted PIs > NFV & APV > IDV > ATV; NNRTI: EFV more common than NVP; NRTI: d4T most common
Monitoring: Fasting lipid profile at baseline, 3–6 months after starting new regimen, then annually or more frequently if indicated (in high-risk patients, or patients with abnormal baseline levels); Follow HIVMA/ACTG guidelines for management; Assess cardiac risk factor
Treatment: Lifestyle modification: diet, exercise, and/or smoking cessation; Switching to agents with less propensity for causing hyperlipidemia; Pharmacologic Management: total cholesterol, LDL, TG 200–500mg/dL: “statins” – pravastatin or atorvastatin; TG >500mg/dL: gemfibrozil or micronized fenofibrate. Use non-PI, non-d4T based regimen; Use ATV-based regimen.

**Insulin Resistance/ Diabetes Mellitus**

Antiretroviral: All PIs
Onset: weeks to months after beginning of therapy
Presentation: Polyuria, polydipsia, polyphagia, fatigue, weakness; exacerbation of hyperglycemia in patients with underlying diabetes
Prevalence: Up to 3%–5% of patients developed diabetes in some series
Monitoring: Fasting blood glucose 1–3 months after starting new regimen, then at least every 3–6 months
Treatment: Diet and exercise; use PI-sparing regimens, consider switching to an NNRTI-based regimen; Metformin; “Glitazones”; Sulfonylurea; Insulin

Adapted from Table 18b, HHS guidelines January 29, 2008 [4].

**Table 3:** Cardiovascular Medications that should not be Used with HIV Drugs

HIV Medication	Cardiovascular Medication
Atazanavir	Bepridil, lovastatin, simvastatin
Darunavir/ritonavir	Lovastatin, simvastatin
Fosamprenavir	Bepridil, lovastatin, simvastatin
Indinavir	Amiodarone, lovastatin, simvastatin
Lopinavir/ritonavir	Flecainide, propafenone, lovastatin, simvastatin
Nelfinavir	Lovastatin, simvastatin
Ritonavir	Bepridil, flecainide, propafenone, quinidine, lovastatin, simvastatin
Saquinavir/ritonavir	Lovastatin, simvastatin
Tipranavir/ritonavir	Bepridil, flecanide, propafenone, quinidine, amiodarone, lovastatin, simvastatin

Adapted from Table 21, HHS guidelines, January 29, 2008 [4].

**Table 4:** Drug interactions Between Statins and ARVs Requiring Caution, Monitoring for Toxicity, and Possibly Dose Adjustment

ARV	Lipid Lowering Agent†		
	Atorvastatin	Pravastatin	Simvastatin or Lovastatin
Atazanavir	Levels may increase. Start with 10 mg.	No data.	Contraindicated.
Fosamprenavir	AUC increased 150%. Start with 10 mg.	No data.	Contraindicated.
Lopinavir/r	AUC increased 5.9 fold. Start with 10 mg.	AUC increased 33%, no dose adjustment necessary.	Contraindicated.
Darunavir/r	10 mg dose gives 40mg exposure. Start with 10 mg.	AUC increased 81%, but up to 5 fold. Use lowest starting dose.	Contraindicated.
Nelfinavir	AUC increased 74%. Start with 10 mg.	No data.	Contraindicated.
Saquinavir/r	Levels increased 450%. Start with 10 mg.	Levels decreased 50%. Use standard dose, adjust based on lipids.	Contraindicated.
Tipranavir/r	AUC increased 9 fold. Start with 10 mg.	No data.	Contraindicated.
Efavirenz	AUC decreased 43%. Start with 10 mg, adjust dose based on lipid responses.	Levels decreased 40%. Use standard dose, adjust dose based on lipids.	Simvastatin AUC decreased by 58%. Adjust based on lipids.
Etravirine	Decreased AUC 37%. Start with 10 mg, adjust dose based on lipid response.	No change for pravastatin, but increased fluvastatin levels may require dose adjustment.	Decreased levels.
Nevirapine	No data.	No data.	No data.

Adapted from Table 22a. HHS guidelines, January 29, 2008 [28].

† Standard dose rosuvastatin can be used with all ARVs.
